# Contemporary breast cancer chemotherapy leads to persistent late right ventricular myocardial dysfunction: a prospective multi-centre study

**DOI:** 10.1186/1532-429X-15-S1-P164

**Published:** 2013-01-30

**Authors:** Suchi Grover, Carmine DePasquale, Govindarajan Srinivasan, Darryl P Leong, Adhiraj Chakrabarty, Kerry A Cheong, Rohit Joshi, Amy Penhall, Majo Joseph, Bogda Koczwara, Joseph Selvanayagam

**Affiliations:** 1 Flinders Medical Centre, Bedford Park, SA, Australia; 2 Flinders University, Adelaide, SA, Australia; 3 Oncology, Flinders Medical Centre, Adelaide, SA, Australia; 4 Adelaide Cancer Centre, Adelaide, SA, Australia; 5 Lyell McEwin Hospital, Adelaide, SA, Australia

## Background

Previous studies evaluating cardiac effects of chemotherapy have focussed on the left ventricle (LV) and largely been retrospective. Although right ventricle (RV) systolic dysfunction is an adverse prognostic marker in cardiomyopathy states, the RV effects of chemotherapy are not well defined.

## Methods

Thirty six breast cancer patients undergoing chemotherapy (26 anthracyline, 10 tranztumab) underwent serial CMR imaging (for LV and RV volumes/ejection fraction) and echocardiography for tricuspid annular systolic plane excursion (TAPSE) at baseline, 1, 3 and 12 months. RV impairment following chemotherapy was defined as an reduction of CMR RVEF below normal range (defined as 50%)1 and/or an absolute reduction of RVEF of at least 5% from baseline.

## Results

Significant changes in CMR volumes and systolic function were observed in both the ventricles (table). Although LV function decreased, EF remained above the lower limit of normal (defined as 57%1) in all patients. RV function changes were of greater magnitude. 23% of patients had significant RV impairment at 3 months and 81% had RV impairment evident at 12 months (as defined above). TAPSE decreased from 23±3 to 21.1±3; p<0.001 at 3 months. Patients that received anthracyclines were more likely to have significant RV impairment at 12 months, p value <0.005.

**Table 1:** Functional changes following chemotherapy

	LVEDVI	LVESVI	LVEF	LVMM	RVEDVI	RVESVI	RVEF
Baseline (n=33)	63±12	17±6	72±6	48±9	66±14	25±8	63±8
1 month (n=33)	64±11	20±6**	69±6**	50±9	69±15	27±8*	60±6*
3 mths (n=33)	65±12	21±7**	68±6**	48±9	67±13	28±7**	58±6**
12 mths (n=21)	73±14**	26±10**	65±6**	40±6	71±11*	35±7**	51±9*

*p <0.05 **p<0.001

## Conclusions

RV functional changes can be detected both early and late following current chemotherapy regimes using CMR and modern echocardiographic techniques. Although anthracyclines likely causes biventricular dysfunction, in our study they were more likely to cause persistent RV impairment at 12 months. These findings support routine surveillance of RV function in contemporary breast cancer chemotherapy.

## Funding

nil
